# Arabic gum ameliorates systemic modulation in Alloxan monohydrate-induced diabetic rats

**DOI:** 10.1038/s41598-023-31897-x

**Published:** 2023-03-27

**Authors:** Rasha Mohammed Ibrahim, Hemmat Mansour Abdelhafez, Sawsan Abd EL-Maksoud EL-Shamy, Fatma Ahmed Eid, Alya Mashaal

**Affiliations:** 1 Cytochemistry and Histology, Zoology and Entomology Department, Faculty of Science (for Girls), Al-Azhar University, Nasr City, Cairo, 11865 Egypt; 2 Biology Department, Basic Science Centre, Misr University for Science and Technology, 6th of October, Egypt; 3 Immunology, Zoology and Entomology Department, Faculty of Science (for Girls), Al-Azhar University, Cairo, 11865 Egypt

**Keywords:** Biochemistry, Cell biology, Immunology, Zoology, Diseases

## Abstract

Medicinal plants are considered an alternative therapy for diabetes mellitus as they regulate glucose levels. Moreover, a variety of plants offer a rich source of bioactive compounds that have potent pharmacological effects without any negative side effects. The present study aimed to clarify the effects of Arabic gum/Gum Acacia (GA) on the biochemical, histopathological, and immunohistochemical changes observed in diabetic rats. Further, the anti-inflammatory activity of GA in response to diabetes, through inflammatory mediators analysis. Male rats were divided into four groups: untreated control, diabetic, Arabic gum-treated, and Arabic gum-treated diabetic rats. Diabetes was induced using alloxan. Animals were sacrificed after 7 and 21 days of treatment with Arabic gum. Body weight, blood and pancreas tissue samples were collected for analysis. Alloxan injection significantly decreased body weight, increased glucose levels, decreased insulin levels, and caused depletion of islets of Langerhans and β-cell damage in the pancreas. Arabic gum treatment of diabetic rats significantly increased body weight, decreased serum glucose levels, increased insulin levels, exerts anti-inflammatory effect, and improved the pancreas tissue structure. Arabic gum has beneficial pharmacological effects in diabetic rats; therefore, it might be employed as diabetic therapy to reduce the hyperglycemic damage and may be applicable for many autoimmune and inflammatory diseases treatment. Further, the new bioactive substances, such as medications made from plants, have larger safety margins, and can be used for a longer period of time.

## Introduction

Diabetes mellitus (DM) is a chronic disorder affecting carbohydrate metabolism and involving hyperglycemia, as a result of deficiencies in insulin release, action, or even both. These range from defects that lead to resistance to insulin action to autoimmune destruction of the pancreatic β-cells with subsequent insulin shortage. It can lead to a series of complications and consequently, to injuries in various tissues and poor quality of life^1^. DM is among the 10 most frequent causes of death worldwide. Therefore, DM poses a unique and significant threat to millions of people over the globe^2^. Epidemiological studies have shown that diabetes is the chronic disease with the highest incidence worldwide^3^. The global prevalence of diabetes is 2.8% among all age groups and is predicted to increase annually to reach a value as high as 4.4% in 2030^4^. Type-1 DM (T1DM) is a chronic autoimmune illness categorized by the insidious and unremitting breakdown of β-cells^5^. This illness is caused by immune system dysfunction, which is mostly driven by T helper 1 (Th1) cells. Additionally, immune cells are activated and infiltrate the islets, resulting in the destruction of pancreatic β-cells and overt hyperglycemia^6^. Hyperglycemia produces reactive oxygen species (ROS) that induce cell damage through different pathways^7^, which leads to secondary complications of DM^8^. T1DM is characterized by the production of cytokines such interferon (IFN), tumor necrosis factor (TNF), and IL-1 by infiltrating immune cells. The transcription factors NF-B and STAT-1 are in charge of activating the gene networks in β-cells, which causes IL-1 and/or TNF plus IFN to directly damage β-cells and mediate apoptosis. Nitric oxide (NO) and chemokines are produced when NF-κB is activated, and endoplasmic reticulum calcium is depleted^9^.

The alloxan-induced diabetes model has been utilized by a number of authors as a "research tool" to clarify the pathophysiology of the disease, in research concerning diabetes, alloxan is a traditional and one of the most prevalent diabetogenic agents frequently used to evaluate the antidiabetic activity of both pure chemicals and plant extracts^10^. Alloxan is one of the drugs used to induce diabetes experimentally. Through its unique actions suppressing glucokinase activity and ROS generation, has a significant necrotizing effect on β-cells. Therefore, the reduction in the number of β-cells results in insulin deficiency followed by carbohydrate, protein, and fat metabolism dysfunctions^11^.

Despite recent advancements in diabetes care, various complications, including hypoglycemia, cardiovascular risks, increased risk of morbidity^12^, and gastrointestinal disorders^13^, are still associated with medications currently available. Natural products isolated from medicinal plants have been used to prevent and cure various diseases, such as cancers, DM, heart diseases, and high blood pressure^14^. Medicinal plants are a tremendous resource to produce medicines. Indeed, medicinal plants have low toxicity and excellent biological activities and economic viability^15^. Antidiabetic plants are used to treat diabetes by reducing the amount of glucose in the blood without inducing pain or other complications^16^. Therefore, despite the worrying statistics on diabetes from organizations such as the World Health Organization, International Diabetes Federation, and American Diabetes Association, with early detection and the right care, DM can be managed, and its complications avoided^2^.

Arabic gum/Gum Acacia (GA) is an edible, dried sticky exudate derived from *Acacia seyal* or *Acacia senegal* stems and branches and is rich in non-viscous soluble fibers^17^. GA consists primarily of two entities, i.e., oligosaccharides and arabinogalactan, which contribute to approximately 97% of GA's total composition. Proteins represent less than 3% of GA^18^. GA can act as a natural fiber to raise the levels of short-chain fatty acids, which have immunomodulatory effects to minimize inflammation and provide patients with a high quality of life^19^. Inflammation is a complex biological response mediated by many factors, such as inflammatory cytokines such as TNF-α and IL-10, the others exerting different effects^20^. GA has historically been used for chronic renal disease, stomach pain, and other disorders. Recently, several pharmacological and medical effects of GA have been evidenced including weight loss, antihyperlipidemic, antihypertensive, antibacterial, anticoagulant, antidiabetic, anti-inflammatory, and nephron-protective actions^21^.

## Materials and methods

### Experimental animal feeding and maintenance

All experiments were performed after approval of the protocol by the Research Ethics Committee of the Faculty of Medicine for Girls, Al-Azhar University (NO. 202009312), and the procedures were carried out in compliance with the National Institutes of Health's Guide for the Care and Use of Laboratory Animals (NIH Publication No. 85-23, revised 1996). The design of the experiment was compatible with the ARRIVE guidelines 2.0. A total of 48 adult male Sprague Dawley rats (120–160 g) aged 7 ± 1 weeks were used for the present study. The animals were obtained from the animal house of VACSERA (Cairo-Helwan, Egypt). The rats were housed and kept under regulated temperature, light and adequate ventilation for 2 weeks before the experimental work. The rats had access to food and water ad libitum and were fed a normal diet (El-Gomhouria Company, Cairo, Egypt).

### Diabetic model (induction of diabetes)

Alloxan was obtained from El-Gomhouria Company, Cairo, Egypt. After a fasting period of at least 8 h, DM was induced in male rats by the intraperitoneal injection of a single dose of alloxan monohydrate (150 mg/kg body weight), which was dissolved in normal saline^22^. Blood glucose levels were measured using a glucometer 48 h after alloxan injection, and rats with fasting blood glucose levels above 250 mg/dl were classified as diabetic^23^.

### Preparation of GA solution

GA was obtained from El-Gomhouria Company, Cairo, Egypt as a fine powder. GA aqueous solution was prepared freshly each day as described by Gamal El-din et al.^24^ and given orally through a gastric tube at a dosage of 25 gm/kg/day. The dose used in rats was estimated based on the human dose using Paget's formula^25^.

### Experimental design

The rats were randomly and equally distributed into four groups (12 rats in each group) as follows: group 1 contained healthy untreated control rats (C), group 2 diabetic untreated rats (diabetes induced using alloxan and maintained for 21 days) (D), group 3 consisted of rats treated with GA orally (25 mg/kg body weight/day) for 21 days (G), and group 4 contained diabetic rats treated with 25 mg/kg body weight GA per day for 21 days (D + G). The experimental rats (6 rats/group) were sacrificed after 7 and 21 days.

### Blood collection and tissue sampling

After the animals of all groups were anesthetized using isoflurane, blood was collected directly from the retro-orbital plexus^26^. Blood samples were left to coagulate at room temperature and centrifuged at 3000 rpm for 10 min to obtain a clear, non-hemolyzed serum for biochemical analysis. The pancreas was quickly dissected, and pancreas samples were prepared for various histological and immunohistochemical analyses.

### Body weight measurement

The body weight of control and treated rats was measured at initiation and different time intervals of the experiment on days 7 and 21 of treatment.

### Biochemical analyses

Fasting serum glucose was estimated using the enzymatic colorimetric method described by Tietz^27^, whereas serum insulin levels were assayed according to the method of Reeves^28^ using kits of Bio Source Europe S.A. Company.

### Cytokine assays

Sandwich enzyme-linked immunosorbent assay (ELISA) was used to detect tumor necrosis factor-α (Abcam, ab46070) and interleukin-10 (Abcam, ab214566) in serum. The optical density was read on a microtiter plate reader with 450 nm (ELX-808, BioTek Instruments, Winooski, VT, USA).

### Histopathological and immunohistochemical analyses

On days ‏7‏ and ‏21‏, animals from the control and treated groups were sacrificed. The pancreas tissues were quickly removed, fixed for 24 h in 10% neutral formalin, dehydrated, cleared in xylene, and embedded in paraffin wax. 5 μm tissues thickness are stained with hematoxylin and eosin for general histological structure. Mallory’s trichrome staining was used to detect collagen fibers^29^ and standard immunohistochemical methods described by Eissa and Shoman^30^ were applied to detect insulin markers.

### Statistical analysis

The data were processed and analyzed using the SPSS program (Statistical Analyses for Social Science, Version 8). Analysis of variance was employed according to Snedecor and Cochran’s method^31^. Student's *t*-test was used to assess significant differences between treatment means. Data are presented as means ± standard deviations, and P ≤ 0.05 was considered statistically significant.

## Results

### Body weight

The diabetic group exhibited a significant decrease in body weight in the first week and a very highly significant decrease after 21 days, which valued 124.6 ± 8.17 and 116.2 ± 7.66 after 7 and 21 days respectively compared to the findings in the control group (134 ± 7.55–144.6 ± 7.09).

Oral administration of GA alone showed non-significant decrease in the mean values of body weight (133.4 ± 9.20 and 143.2 ± 8.76) on 7 and 21 days post-treatment in comparison with the control group.

**Figure 1:**
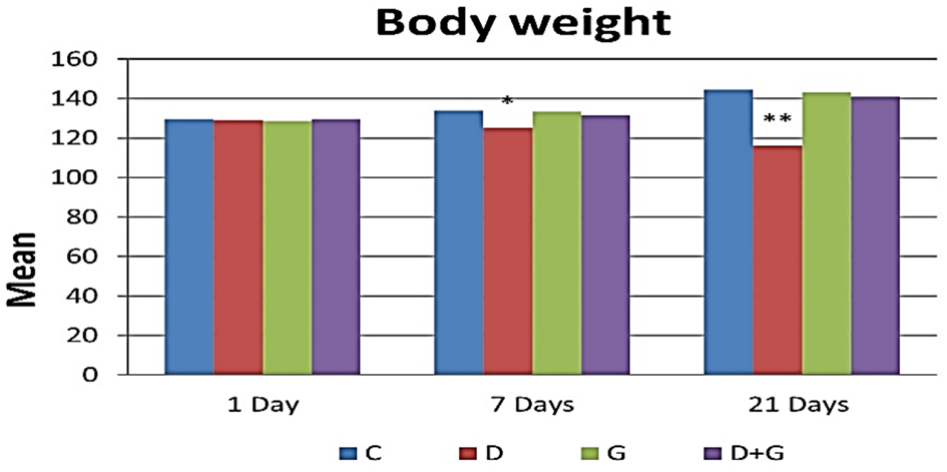
The average of body weight associated with Arabic gum and diabetes-induced rats. Each value is presented as the mean ± standard deviation (SD), n = 6 rats. *Significant difference vs the control group at P < 0.05, **highly significant difference vs the control group at P < 0.01. *C* control group, *D* diabetic group, *G* Arabic Gum group, *D + G* diabetic-Arabic Gum treated group.

Moreover, the diabetic rats which were treated with GA were normalized (131.6 ± 4.45–141 ± 6.08) when compared to diabetic rats (Fig. 1).

### Glucose and insulin levels

**Figure 2:**
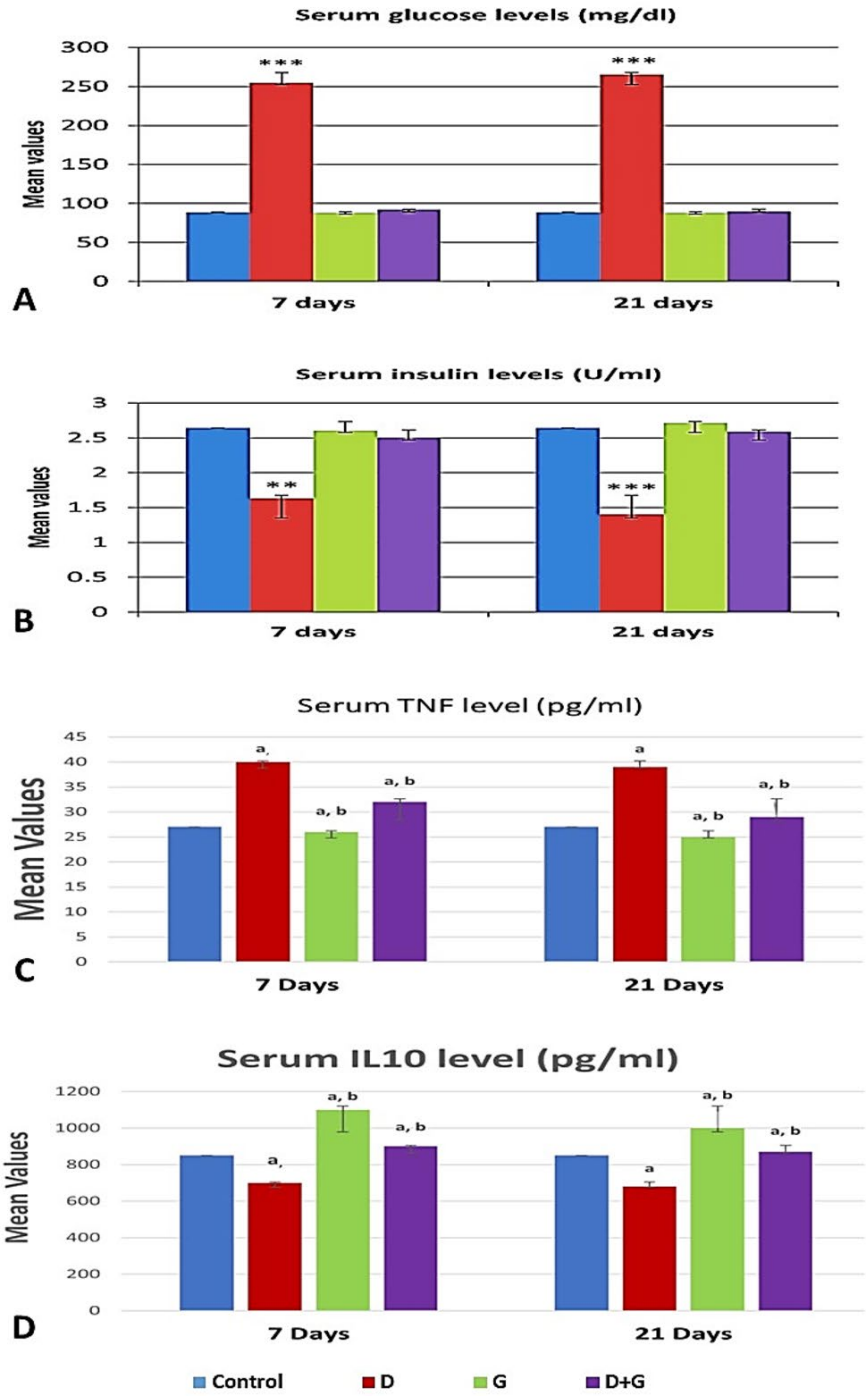
Circulating glucose, insulin, tumor necrosis factor-alpha, and interleukin-10 concentrations associated with Arabic gum and diabetes-induced rats. Each value is presented as the mean ± standard deviation (SD), n = 6 rats. (**A**) Glucose mean values. (**B**) Insulin mean values. (**C**) Tumor necrosis factor-alpha (TNF-α) mean values. (**D**) Interleukin-10 (IL10) mean values. *C* control group, *D* diabetic group, *GA* Arabic Gum group, *D + G* Diabetic-Arabic Gum treated group, *TNF-α* tumor necrosis factor-alpha, *IL-10* interleukin-10. **Highly significant difference vs the control group at P < 0.01. ***very significant difference from the mean of the control group at P < 0.001. Superscripts indicate significance (P < 0.05) where: ^a^denotes significance vs. control group; ^b^denotes significance vs. diabetic group.

The present results showed very highly significant increases (P < 0.001) in the mean values of serum glucose levels of the diabetic group which reached 254.71 ± 7.43 and 265.33 ± 6.92 mg/dl on 7 and 21 days post-treatment respectively as compared to the untreated control group (88.60 ± 2.99 and 88.61 ± 2.98). Accompanied by very highly significant decreases (P < 0.001) in the mean values of serum insulin levels such decreases were 1.63 ± 0.13 and 1.40 ± 0.27 U/ml after 7 and 21 day as compared to the control group (2.64 ± 0.35 and 2.63 ± 0.34). Oral administration of GA alone showed non-significant changes (P > 0.05) in the mean values of serum glucose (88.17 ± 1.79 and 87.87 ± 1.05) and insulin (2.60 ± 0.22 and 2.71 ± 0.22) levels on 7 and 21 days post-treatment in comparison with the control group. Additionally, treatment with GA post-alloxan injection significantly decreased serum glucose levels (92.18 ± 2.58 and 89.70 ± 4.52) on the 7 and 21 day post-treatment respectively) and it significantly increased insulin levels (2.49 ± 0.21 and 2.59 ± 0.22 U/ml at 7 and 21 days post-treatment respectively) as compared with the diabetic groups (Fig. 2A,B).

### Systemic inflammation

The effect of GA treatment on diabetic/non-diabetic rats with circulating inflammatory and anti- inflammatory markers expressed by TNF-α and IL-10 cytokines was shown in Fig. 2. TNF-α concentration of Arabic gum-diabetic group (32 ± 0.84 and 29 ± 0.82) was significantly decreased compared to diabetic group (40 ± 0.63 and 39 ± 0.89) at time intervals 7 and 21 days respectively, in contrast to its expression compared to control (27 ± 0.63 and 27 ± 0.62) which significantly increased. IL-10 was significantly increased (900 ± 1.41 and 870 ± 1.09) compared to both control (850 ± 1.26 and 850 ± 1.16) and diabetic groups (700 ± 1.27 and 680 ± 2.09) at experiment durations respectively (Fig. 2C,D).

### Histological analysis

**Figure 3:**
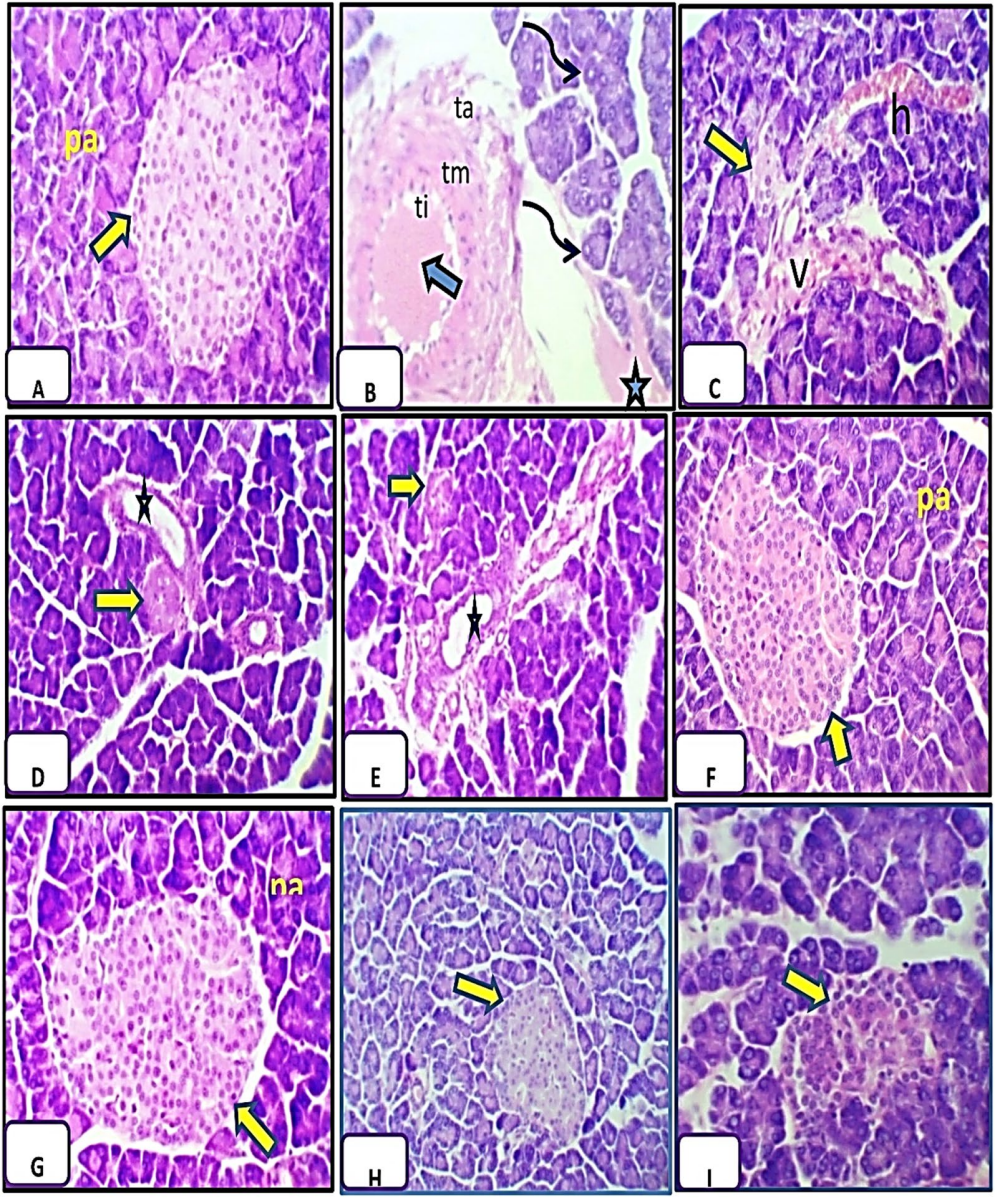
Pancreatic tissue sections from control and treated rats stained with hematoxylin and eosin. (**A**) Normal appearance of islets of Langerhans (arrow) and pancreatic acini (pa) in the control group. (**B**,**C**) Representative pancreas sections from diabetic rats showing hypertrophied nuclei of tunica intima (ti), faintly stained nuclei of tunica media (tm), distorted connective tissue of tunica adventitia (ta), hemolyzed blood cells (arrow), edematous area (star), hemorrhagic area (h), and atrophied islet of Langerhans (arrow) and degenerative changes in the nuclei of the acini like absence of some of them (black curved arrow) 7 days after alloxan injection. (**D**,**E**) Representative pancreas sections from diabetic rats showing distorted islets of Langerhans (arrows), interlobular ducts (stars) containing hemolyzed blood cells 21 days after alloxan injection. (**F**,**G**) Representative pancreas sections from rats treated with Arabic gum (GA) showing normal islets of Langerhans (arrows) and pancreatic acini (Pa) after 7 (**F**) and 21 days (**G**) of treatment. (**H**,**I**) Representative pancreas sections from rats treated with alloxan and GA showing an improved architecture of islets of Langerhans (arrows). Magnification: ×400 in (**A**,**B**,**F**,**G**,**I**) and ×250 in (**C**,**D**,**E**,**H**).

**Figure 4:**
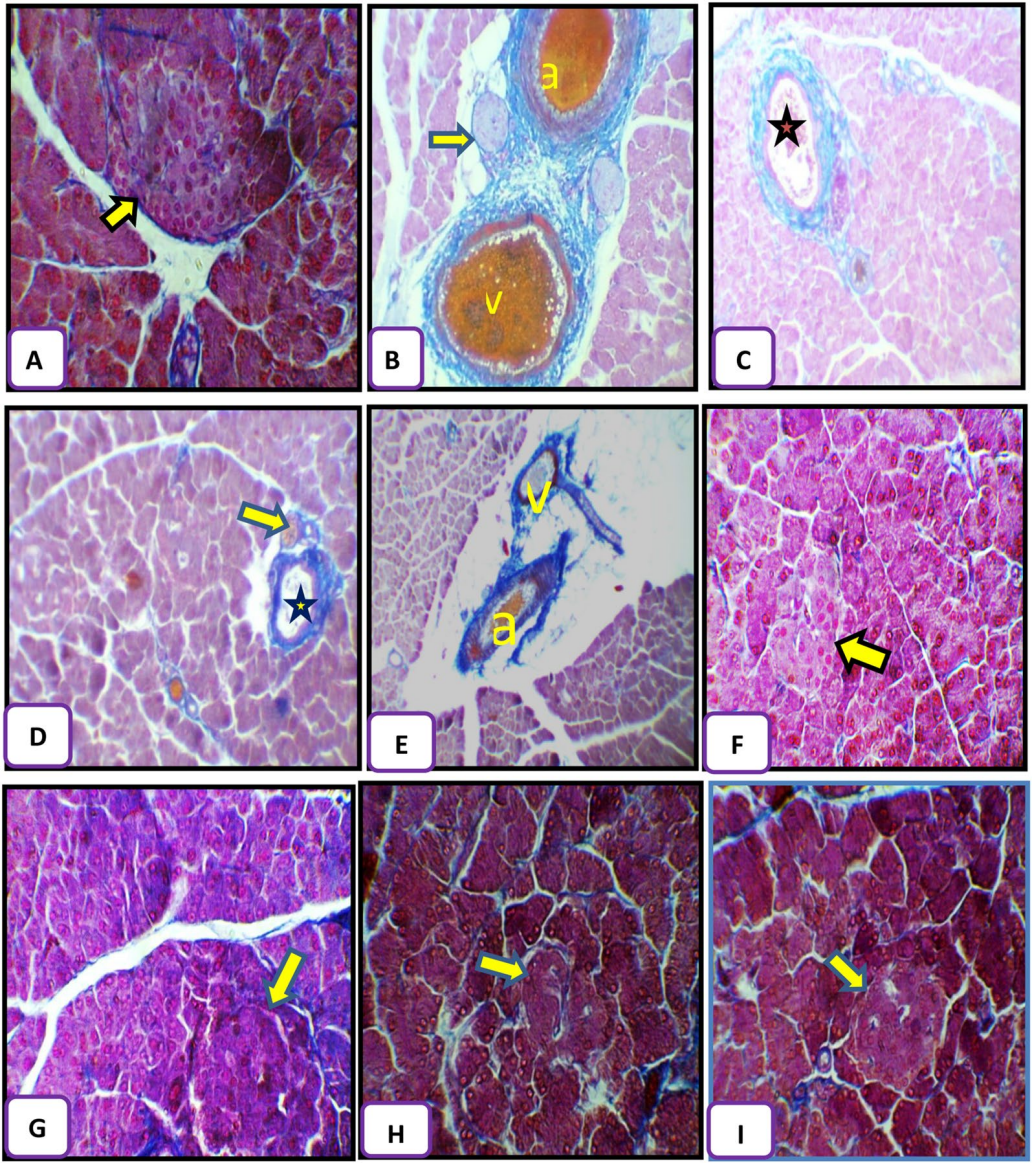
Pancreatic tissue sections from control and treated rats after Mallory's trichrome staining. (**A**) Representative control section showing normal distribution of collagen fibers (arrow). (**B**,**C**) Pancreatic tissue sections from diabetic rats showing highly increased amounts of collagen fibers in the artery (a), vein (v) and interlobular duct (star) and atrophied islets of Langerhans (arrow) 7 days after alloxan treatment. (**D**,**E**) Pancreatic tissue sections from diabetic rats showing higher numbers of collagen fibers in the interlobular duct (star), islets of Langerhans (arrow), the artery (a), and the vein (v) 21 days after alloxan treatment. (**F**,**G**) Pancreatic tissue sections from rats treated with Arabic gum (GA) showing thin collagen fibers (arrows) after 7 and 21 days of treatment. (**H**,**I**) Pancreatic tissue sections from diabetic rats treated with GA (D + G group) showing a slightly increased number of collagen fibers (arrows) after 7 and 21 days of treatment, respectively. Magnification ×250 in (**B**–**D**), ×200 in (**E**), and ×400 in (**A**,**F**,**G**,**H**).

The pancreas tissues were stained with H&E and Mallory’s trichrome stains to assess the effect of GA on pancreas damage induced by alloxan injection. The control group displays a normal appearance of islets of Langerhans and the pancreatic acini with their triangular cells which contained basal rounded nuclei (Fig. 3A) with normal distribution of collagen fibres around islets of Langerhans and the pancreatic acini (Fig. 4A).

The histological examination of the pancreas tissues showed that alloxan injection after 7 days caused a highly thickened arterial wall with hypertrophied nuclei of endothelial lining of tunica intima, faintly stained nuclei of muscle cells of tunica media and highly distorted connective tissue of tunica adventitia, hemolyzed blood cells inside the artery in addition to the presence of edematous areas around the pancreatic acini, also degenerative changes in the nuclei of the acini were detected such as absence some of them (Fig. 3B), further, highly elongated and ruptured wall of the venule which contained hemolyzed blood cells inside it with large hemorrhagic areas were also detected at 7 days post alloxan treatment (Fig. 3C). Concerning the distribution of collagen fibres in pancreatic tissue of diabetic group after 7 days, highly increased collagen fibres were detected in and around walls of arteries, veins, interlobular duct also in between the acini, around the highly atrophied islets of Langerhans and around the pancreatic tissue (Fig. 4B,C).

By the end of 21 days following alloxan injection, highly atrophied and distorted islets of Langerhans were detected, most of the different cells inside it disappeared, but a few pyknotic nuclei were detected, interlobular ducts contained hemolyzed blood cells inside them with a highly stratified wall of their cuboidal cells (Fig. 3D,E). Also, highly increased collagen fibres were observed in and around the interlobular duct around the islets of Langerhans, in between the pancreatic acini, in walls of the artery, vein and around them (Fig. 4D,E).

Rats administrated GA alone showed normal histological appearance of the pancreatic tissue after seven (Fig. 3F) and 21 days (Fig. 3G) with thin collagen fibres supported islets of Langerhans, interlobular duct and in between the pancreatic acini after seven (Fig. 4F) and 21 days (Fig. 4G) post-treatment.

Pancreas sections of rats administrated GA for 21 days post-alloxan injection and examined after 7 and 21 days showed improvement architecture of islets of Langerhans and the pancreatic acini after 7 days (Fig. 3H) as compared to the diabetic group with slightly increased collagen fibres deposition in between the exocrine acini and around the islets of Langerhans (Fig. 4H) as compared to the control group. As shown in Fig. 3I islets of Langerhans and exocrine acini resumed their normal structure after 21 days with thin scattered collagen fibres supported walls of the artery and vein, around the islets of Langerhans and in between the pancreatic acini (Fig. 4I).

### The immunohistochemical results

**Figure 5:**
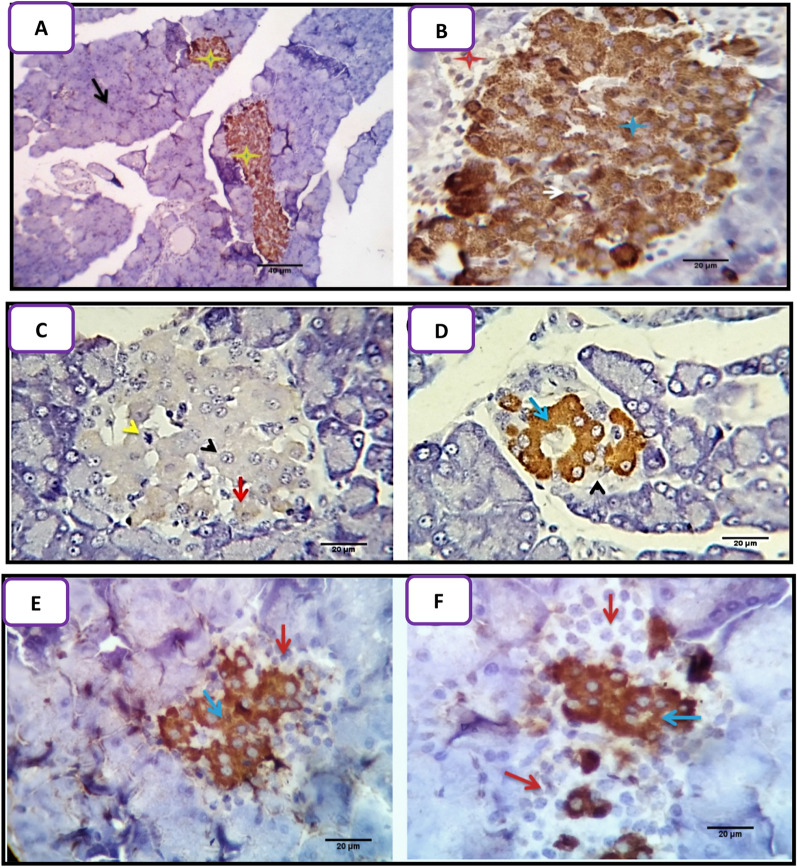
Detection of insulin in the pancreatic tissues of the control and diabetic groups. (**A**,**B**) Insulin-positive cytoplasmic staining in β-cells of the islets of Langerhans (yellow and blue stars). In contrast, α-(red star) and δ-cells (white arrow) were not stained. Acini of the exocrine pancreas were visible (black arrow). (**C**,**D**) Strong insulin-positive staining of islet β-cells (red and blue arrows) 7 days after alloxan injection. The remaining cells were not stained (yellow and black arrowheads). **(E**,**F**) Very intense insulin-positive staining of islet β-cells (blue arrows) 21 days after alloxan treatment. The remaining cells were not stained (red arrows). Scale bars 40 µm in (**A)** and 20 µm in (**B**–**F**).

Immunohistochemical examination of non-diabetic non-treated rats for detection of insulin marker revealed active positive cytoplasmic stain ability of almost beta cells of islets of Langerhans. Alpha and delta cells were negatively stained (Fig. 5A,B).

The diabetic rat’s pancreas on 7 days post-alloxan-injection showed 10–35% high power field (HPF) positively reacted and stained cells with insulin diabetic marker (some sections showed weak staining reactivity), the remaining cells 65–90% HPF were negatively stained. Other islet cells were also unstainable (Fig. 5C,D). Meanwhile, 21 days post-alloxan injection diabetic rat’s pancreas revealed 20–45% HPF positively reacted and stained cells with insulin diabetic marker, the remaining cells 55–80% HPF were negatively stained (Fig. 5E,F).

**Figure 6:**
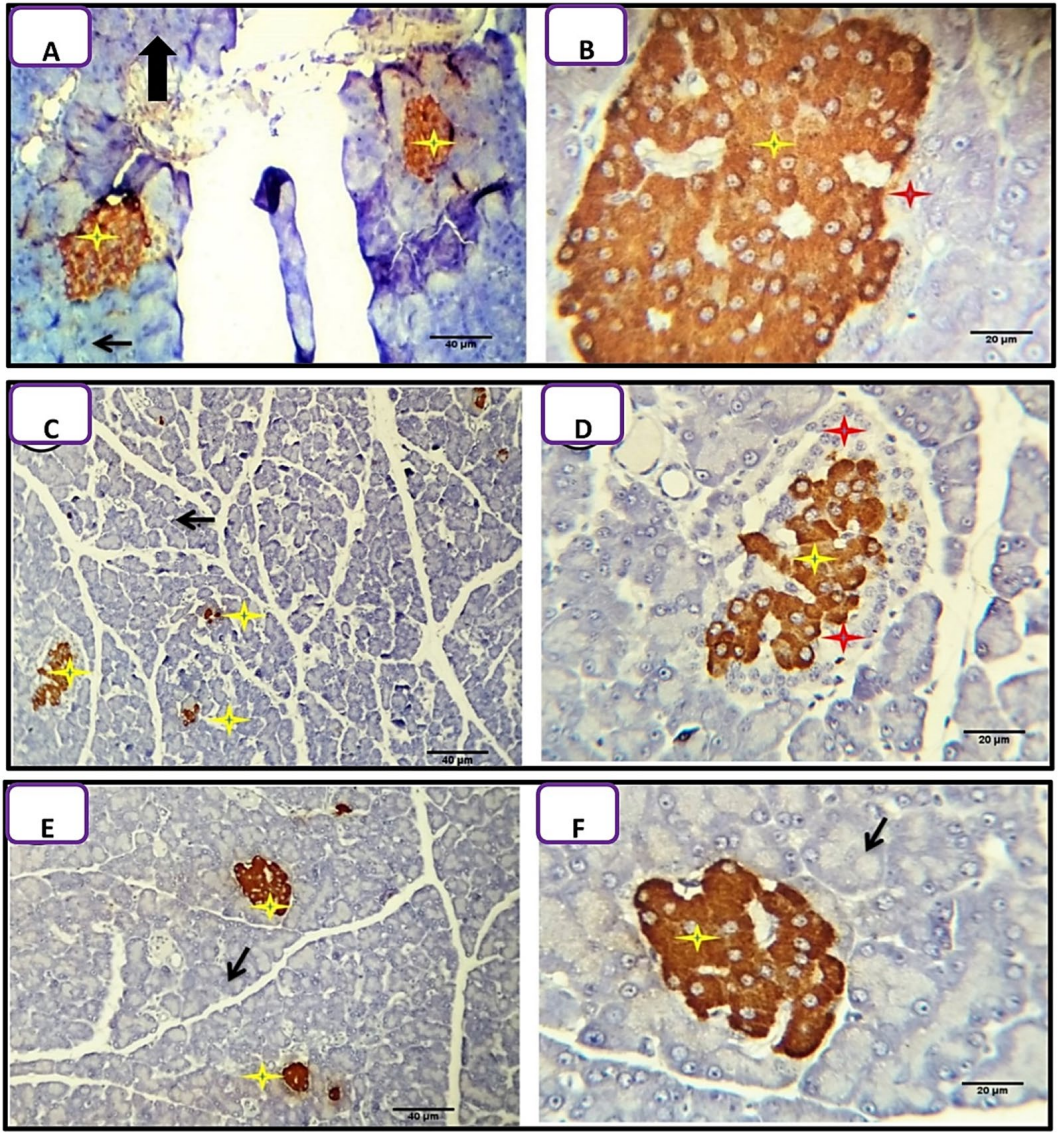
Detection of insulin in the pancreatic tissues of Arabic gum-treated and Arabic gum/diabetic rats. (**A**,**B**) Positive insulin staining of most β-cells (yellow stars) after 7 and 21 days of treatment with Arabic gum. The α-cells (red stars) and acini of the exocrine pancreas (black arrow) were not stained. **(C**,**D**) Moderate immunostaining of β-cells with brown intracytoplasmic granules (yellow stars), whereas α-cells were not stained (red stars) in diabetic rats treated with Arabic gum (D + G group) for 7 days. (**E**,**F**) Most of the islet β-cells were positively stained in diabetic rats treated with Arabic gum for 21 days. Scale bars 40 µm in (**A**,**C**,**E**) and 20 µm in (**B**,**D**,**F**).

Most of the beta cells of the islets of Langerhans (95–100% HPF) of Arabic gum treated groups were positively stained for the insulin marker in most of the examined sections. Very few cells, mostly of Alpha type were unstained after 7 (Fig. 6A) and 21 days (Fig. 6B).

Sections from the pancreas of alloxan-induced diabetic rats treated with Arabic gum 7 days post-alloxan-injection showed partial enhancement of beta cells for insulin marker staining reactivity as the positively stained cells were ranged from 60 to 70% HPF. Most of the cells were intensely stained with the presence of characteristic brown intra cytoplasmic granules (Fig. 6C,D). Meanwhile, sections from the pancreas of alloxan-induced diabetic rats treated with Arabic gum 21-day post—alloxan injection showed complete enhancement of beta cells for insulin marker staining reactivity as the positively stained cells were ranged from 95 to 100% HPF, most of the cells were intensely stained with the presence of characteristic brown intracytoplasmic granules (Fig. 6E,F).

## Discussion

Insufficient insulin frequently caused fast, considerable weight loss in diabetes mellitus patients. As a result, glucose provides the body with a very insufficient amount of energy, causing it to burn through its fat and muscle reserves and cause weight loss^32^. Administration of GA in this study improved the weight gain against alloxan diabetic rats. High dietary fibre intake, including GA, is linked to favorable effects on fat metabolism. Dietary fibre enhances feelings of fullness and satiety, modifies the glycemic index, influences stomach emptying and gut hormone release, and hence aids in weight management^33^.

The present results showed an elevation in glucose serum concentration and a reduction in insulin levels of diabetic rats compared to untreated group. The severe hyperglycemia in diabetic rats reported in the present study may be a direct consequence of the hypoinsulinemia induced by the specific cytotoxicity of alloxan toward β-cells resulting in β-cell vacuolation. Alloxan directly affects the permeability of the cell membrane by triggering ionic pump failure and increasing cell size, thus, inhibiting intracellular energy generation. This may cause a reduction in ATP levels, an inhibition of insulin production, and cell necrosis^34^.

Lenzen reported that alloxan inhibits glucose release induced by insulin by specifically inhibiting glucokinase activity and triggers an insulin-dependent diabetes condition by stimulating the generation of ROS through a cyclic reaction with its reduction product, dialuric acid^13^. Islet cells exposed to alloxan produce substantially higher levels of peroxynitrite, nitric oxide, and ROS and exhibit significantly increased lipid peroxidation, decreased cell viability, and increased mitochondrial membrane potentials. Alloxan induces extreme oxidative and cytotoxic stress in islets, likely resulting in an impaired cell ability to release insulin^35^.

In the current study, GA exerts anti-inflammatory effect similarly reported by Ali et al.^36^. Inflammation and oxidative stress mediate various pathophysiological features in many diseases^37^, factors that can control and reduce these features may be effective as prophylactic or treated agent of these diseases. The balance between TNF-α and the IL-10 incidence play an important role in keeping the inflammation homeostasis^38^. Triggering of these cytokines leads to inflammation, resulting in the production of even more reactive oxygen species, which can contribute to even more oxidative stress, generating a feedback loop^39^. The ability of GA to reduce inflammatory cytokines and oxidative stress mediators, which are markedly diminished by its administration, may be one of the ways by which it controls diabetes, these finding are in parallel to those of Ali et al.^40^.

In the present study, the mean serum levels of glucose and insulin of GA alone or GA-diabetic group were not significantly different from the control group. The improvement of serum glucose levels in diabetic rats treated with GA is supported by the results of Babio et al*.* who reported important physiological benefits of dietary fibers, which decrease postprandial glucose and lower cholesterol plasma concentrations^41^. In addition, dietary fibers were shown to have potential hypoglycemic effects by decreasing blood glucose levels in a diabetic rabbit model^42^.

The present data also agree with the results from Hegazy et al. who reported that oral administration of two distinct doses (100 and 200 mg/kg) of *Acacia arabica* extract to diabetic rats for 21 days decreased the elevation in serum glucose and insulin, diminished insulin resistance, and enhanced lipid metabolism^43^. The same authors added that all these results were secondary to the elevated levels of insulin. Another work also showed that food supplementation with GA (10 g/day for 16 weeks) in prediabetic and diabetic subjects significantly reduces fasting blood sugar and glycated hemoglobin levels^44^. Finally, the present results are also supported by the study by Al Zaabi et al*.* who reported that GA in drinking water (15% w/w) prevents, at least partly, streptozotocin's effect by stimulating insulin release and increasing insulin resistance^45^.

The pancreas of the diabetic rats contained highly atrophied islets of Langerhans and exhibited a hypocellularity affecting the different cell types. The present findings are supported by studies conducted by El-Esawy et al. who reported a degeneration, atrophy, and vacuolization in the islets of Langerhans and β-cells, a reduced number of β-cells, and loss of normal islet structure in diabetic animals^46^. Experimentally, alloxan causes major injuries to pancreatic β-cells by producing ROS, leading to the induction of diabetes in animals^47^. Various mechanisms have been suggested to explain islet cell injuries, especially β-cell damage, in T2DM. These include increased oxidative stress, enhanced metabolic stress, increased endoplasmic reticulum stress, activation of inflammatory pathways, and islet amyloid polypeptide toxic accumulation. In all cases, the result is a β-cell dedifferentiation and apoptosis^48^.

Tangvarasittichai observed that hyperglycemia is the cause of biochemical processes such as oxidative stress, low-grade inflammation, and apoptosis^49^, and that these biochemical changes precipitated the development of insulin resistance and the degradation of β-cells. Additionally, the average area occupied by insulin immunoreactivity in immunohistochemical experiments is greatly diminished in diabetic animal models^50^. Moreover, inflammatory and apoptotic pathways lead to β-cell damage in a rat model of T2DM induced by high-fat diet and STZ as confirmed by inducible nitric oxides and caspase-3 immunohistochemistry.

The pancreatic tissues of the rats in the current investigation that received GA were normal; GA is a recognized safe direct food additive and has no known genotoxic effects^51^. Furthermore, developmental toxicity has not been associated with intraperitoneal or oral administration of GA^52^, and no carcinogenic effects of GA were reported^53^. Although GA is indigestible to both humans and animals, it is fermented in the colon to produce short-chain fatty acids, which have a wide range of potential health advantages^54^.

We observed signs of improvements in histopathological modifications and the state of the islets of Langerhans and pancreatic acini observed in diabetic animals post GA treatment. These data agree with those described previously by Eliza et al. who proposed that* A. arabica* extracts have an insulin-like action by increasing glucose absorption into the muscle and adipose tissues and by inhibiting hepatic gluconeogenesis^55^. It was also suggested that *A. arabica* exerts its hypoglycemic effect by activating insulin receptors^56^. Yasir et al*.* showed that, although there are other mechanisms, *A. arabica* main mechanism of action occurs through the revitalization and maybe the regeneration of the damaged β-cells as suggested by the increased serum levels of insulin in diabetic rats treated with *A. arabica* extract^57^. This mechanism can be explained by the antioxidant properties of *A. arabica* extracts, which are evidenced by a decreased malondialdehyde concentration and increased coenzyme-Q10 levels^58^. GA can act as a hypoglycemic agent by causing the production of insulin from pancreatic β-cells in healthy rabbits^59^. Plants have hypoglycemic, antihyperlipidemic, and antioxidant actions because they are important sources of flavonoids, gallotannins, amino acids, and other related polyphenols^60^. Many components, including polyphenols, tannins, and flavonoids (such as quercetin), are found in *A. arabica*. Tannins were shown to improve the function of pancreatic β-cells and promote insulin secretion^61^. Quercetin is an antioxidant that functions through several pathways, including the scavenging of oxygen radicals; therefore, it protects against lipid peroxidation and chelation of metal ions^58^. These antioxidant substances may underlie the antidiabetic effects of *A. arabica*^62^.

## Conclusion

Arabic gum has effective pharmacological role in diabetic rats’ recovery; in addition, GA administration modulate pro- and anti-inflammatory pathways and exert protective effect. Tackling all findings together, GA might be employed as diabetic therapy to reduce inflammation and hyperglycemic damage. Our manuscript creates a paradigm for future studies to investigate gene expression/inflammatory cells markers that modulate and regulate the mechanism action and relationship between GA and different cells reaction, to obtain optimized therapeutical effect of GA.

## Data Availability

Data will be available when requested from the corresponding author.
